# Efficacy of Standard Versus Enhanced Features in a Web-Based Commercial Weight-Loss Program for Obese Adults, Part 2: Randomized Controlled Trial

**DOI:** 10.2196/jmir.2626

**Published:** 2013-07-22

**Authors:** Clare E Collins, Philip J Morgan, Melinda J Hutchesson, Robin Callister

**Affiliations:** ^1^Priority Research Centre in Nutrition and Physical Activity, Nutrition and DieteticsSchool of Health Sciences, Faculty of HealthUniversity of NewcastleCallaghanAustralia; ^2^Priority Research Centre in Nutrition and Physical ActivitySchool of Education, Faculty of Education & ArtsUniversity of NewcastleCallaghanAustralia; ^3^Priority Research Centre in Nutrition and Physical ActivitySchool of Biomedical Sciences & Pharmacy, Faculty of HealthUniversity of NewcastleCallaghanAustralia

**Keywords:** intervention studies, weight loss, Internet, randomized controlled trial, reducing diet, telemedicine

## Abstract

**Background:**

Commercial Web-based weight-loss programs are becoming more popular and increasingly refined through the addition of enhanced features, yet few randomized controlled trials (RCTs) have independently and rigorously evaluated the efficacy of these commercial programs or additional features.

**Objective:**

To determine whether overweight and obese adults randomized to an online weight-loss program with additional support features (enhanced) experienced a greater reduction in body mass index (BMI) and increased usage of program features after 12 and 24 weeks compared to those randomized to a standard online version (basic).

**Methods:**

An assessor-blinded RCT comparing 301 adults (male: n=125, 41.5%; mean age: 41.9 years, SD 10.2; mean BMI: 32.2 kg/m^2^, SD 3.9) who were recruited and enrolled offline, and randomly allocated to basic or enhanced versions of a commercially available Web-based weight-loss program for 24 weeks.

**Results:**

Retention at 24 weeks was greater in the enhanced group versus the basic group (basic 68.5%, enhanced 81.0%; *P*=.01). In the intention-to-treat analysis of covariance with imputation using last observation carried forward, after 24 weeks both intervention groups had reductions in key outcomes with no difference between groups: BMI (basic mean –1.1 kg/m^2^, SD 1.5; enhanced mean –1.3 kg/m^2^, SD 2.0; *P*=.29), weight (basic mean –3.3 kg, SD 4.7; enhanced mean –4.0 kg, SD 6.2; *P*=.27), waist circumference (basic mean –3.1 cm, SD 4.6; enhanced mean –4.0 cm, SD 6.2; *P*=.15), and waist-to-height ratio (basic mean –0.02, SD 0.03; enhanced mean –0.02, SD 0.04, *P*=.21). The enhanced group logged in more often at both 12 and 24 weeks, respectively (enhanced 12-week mean 34.1, SD 28.1 and 24-week mean 43.1, SD 34.0 vs basic 12-week mean 24.6, SD 25.5 and 24-week mean 31.8, SD 33.9; *P*=.002).

**Conclusions:**

The addition of personalized e-feedback in the enhanced program provided limited additional benefits compared to a standard commercial Web-based weight-loss program. However, it does support greater retention in the program and greater usage, which was related to weight loss. Further research is required to develop and examine Web-based features that may enhance engagement and outcomes and identify optimal usage patterns to enhance weight loss using Web-based programs.

**Trial Registration:**

Australian New Zealand Clinical Trials Registry (ANZCTR) trial number: ACTRN12610000197033; https://www.anzctr.org.au/Trial/Registration/TrialReview.aspx?id=335159 (Archived by WebCite at http://www.webcitation.org/6HoOMGb8j).

## Introduction

Internationally, obesity rates in adults continue to rise unabated [1]. Effective treatment programs with broad reach are urgently required. Web-based weight-loss programs are an increasingly viable option because most US and Australian households (66% [2] and 72% [3], respectively) have access to broadband Internet, and many adults (61% in the United States) seek information on health, nutrition, and weight loss from the Internet [4].

A systematic review of the effectiveness of Web-based weight loss and maintenance interventions found that these programs can facilitate meaningful weight change [5]. However, it was not possible to determine their overall effectiveness because of the heterogeneity of designs and small number of comparable studies. A meta-analysis of 3 Web-based weight-loss randomized controlled trials (RCTs) that compared online education-only programs with online programs that included enhanced features, such as counseling, automated or therapist feedback, behavioral lessons, self-monitoring, and a bulletin board, found weight loss was increased by 2.2 kg over a 6- to 12-month period [5]. These results are supported by 3 other RCTs which found that the addition of online lessons with daily self-monitoring of weight, eating, and exercise and computer-generated feedback [6], or the addition of peer support [7], or individually tailored action plans [8], resulted in greater weight loss after 24 weeks [6], a trend toward a greater effect size after 12 weeks [7] and greater weight loss [8] compared to an online program without the enhanced features. Krukowski et al [9] have also demonstrated that participant’s usage of feedback components of a Web-based weight-loss program (eg, progress charts) was the most significant predictor of weight loss after 6 months. By contrast, 2 other RCTs found that adding online lessons or a weekly online group chat session to a Web-based weight-loss program was equally effective up to 12 and 16 weeks, respectively, as a Web-based program without these features [6,10]. Further, all these studies were conducted in the United States [6-10]. Additional longer-term studies from other regions of the world are required to evaluate the superiority, or otherwise, of Web-based programs with enhanced features.

Within the currently available online commercial weight-loss programs, there is a large degree of variation across the range of features provided, including blogs, chat rooms, self-monitoring tools for weight, diet, and physical activity, and also differing types and amounts of feedback from generic to tailored information and human e-counseling. To date, the ability of these more personalized enhanced features to facilitate greater weight loss has only had limited evaluation because programs have not tracked use of specific features [11].

We have previously compared the efficacy of a standard commercial Web-based weight-loss program (basic) versus an enhanced version of this Web program that provided additional personalized e-feedback and contact from the provider (enhanced) versus a waitlist control group [12,13]. After 12 weeks, we found both Web-based programs produced significantly greater weight loss and reductions in body mass index (BMI) compared to the waiting list control group, but no differences in the weight-related outcomes were observed between the 2 programs. Part 2 of the study aims to determine whether overweight and obese adults randomized to the enhanced version of the commercial Web-based weight-loss program achieve a larger reduction in BMI and usage of program features compared to those randomized to a standard version of the online program without these features after 24 weeks.

## Methods

This assessor-blinded RCT recruited overweight and obese adults from the Hunter community in New South Wales, Australia, who were enrolled offline in 2009. Eligibility criteria included age 18 to 60 years, BMI 25 to 40 kg/m^2^, not participating in other weight-loss programs, pass a health screen [14], available for in-person assessments, and access to a computer with email and Internet services. Written informed consent was obtained from all participants, and ethical approval obtained from the University of Newcastle Human Ethics Research Committee. The trial conformed to the Consolidated Standards of Reporting Trials (CONSORT)-eHealth Checklist (Multimedia Appendix 1) [15].

### Stratification and Randomization

**Figure 1:**
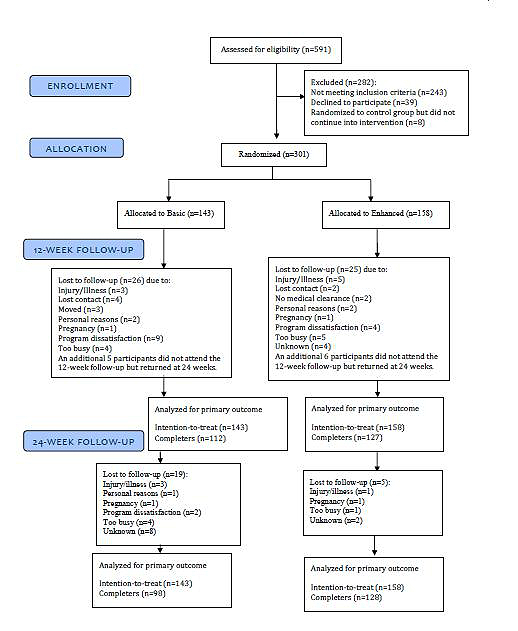
Participant flow.

After baseline assessments were completed, participants were stratified by sex and BMI category (25 to <30, ≥30 to <35, or ≥35 to 40 kg/m^2^) and randomized using a stratified block design to either the standard (basic) Web-based weight-loss program or the same program with additional features (enhanced) (Figure 1). At baseline, participants could also have been randomized to a waitlist control group who were not provided with access to the weight-loss program website. After 12 weeks, participants in the control group were rerandomized into either the basic or enhanced groups and data collected after this rerandomization were included in this analysis. Participants who dropped out before rerandomization to a treatment arm, or achieved their weight-loss goal (≥10% of baseline weight lost) and had, therefore, entered the weight maintenance phase, were also excluded from this analysis.

### Web-Based Weight-Loss Programs (Basic and Enhanced)

Participants were provided with free access to the basic or enhanced version of a commercial Web-based program provided by SP Health Co Pty Ltd in Australia under the name The Biggest Loser Club. The basic program was the version commercially available at the time of the study (2009-2010). Program features are reported in Table 1. The enhanced program contained additional features to the basic program and was provided in a closed test environment. At baseline, participants were given instructions to log in and set up their program details. They were also given a company phone number in case they experienced any difficulties in logging in. Participants did not receive any training on program use to mirror the commercial program engagement experience and increase external validity. Participants were blinded to group allocation and accessed the website using their usual Internet connection.

The 12-week Web-based programs were based on social cognitive theory [17]. Key behavior change mediators targeted included self-efficacy, goal setting, self-monitoring, outcome expectations, and social support. An individualized daily energy intake target to facilitate a weight loss of 0.5 to 1 kg per week was set, as well as a goal weight. Participants were encouraged to self-monitor by reporting their weight or other body measurements via the website or short message service (SMS) text messages once per week and could view graphs and charts to track their progress overtime. They were also encouraged to self-monitor their dietary intake and exercise using an online diary at least 4 days per week. The diary provided automated feedback on daily and weekly energy intake and expenditure, and a weekly summary macronutrient and micronutrient intake compared with recommended targets. Social support was available via a discussion board. Online information was provided weekly (calorie-controlled, low-fat menu plans and grocery lists; physical activity plan based on exercise preferences; educational tips and challenges) which participants were prompted to access via a weekly email newsletter.

At the end of 12 weeks, participants could choose to repeat the same weekly 12-week program or to continue for an additional 12 weeks with content varied based on the season and keeping their entire accumulated personal progress and data.

**Figure 2:**
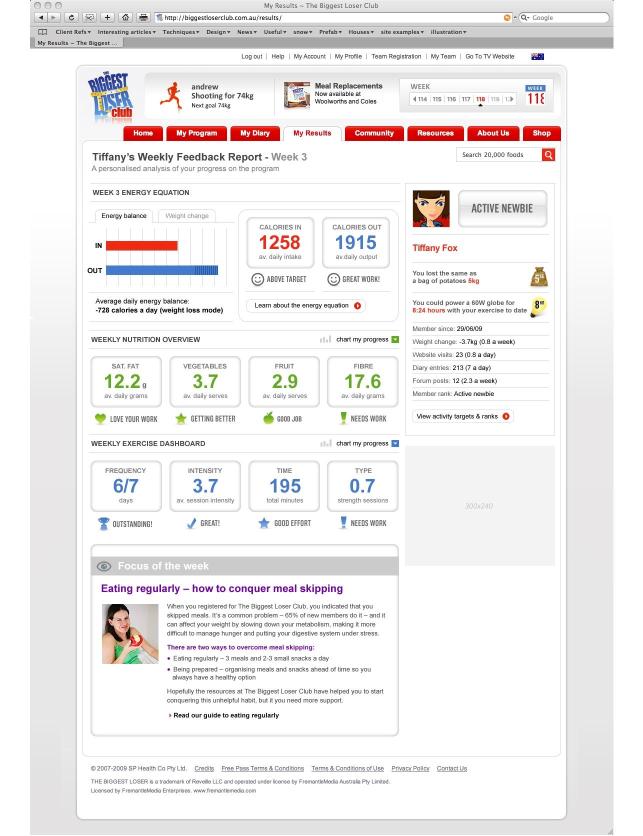
Enhanced groups weekly automated personalized feedback.

The enhanced program included all the basic program features described previously. The additional components were: (1) personalized, system-generated enrollment reports that suggested appropriate weight-loss goals and key behavior changes required for success based on response to a behavioral survey at enrollment; (2) weekly automated system-generated, personalized e-feedback for key elements of diet and physical activity based on diary entries, usage patterns of website features, and level of success with weight loss (Figure 2); (3) an escalating reminder schedule to use the diary, visit the program site, and enter a weekly weight (an initial reminder email, then a SMS text message if there was no response, then a reminder phone call if a weekly weight was still not entered).

**Table 1:** Description of the basic and enhanced commercial Web-based weight-loss programs.

Basic and enhanced	Enhanced only
Participants set weight-loss goals, advised to self-monitor their weight, waist, and hip girths. Encouraged to self-monitor via weekly email and/or short message service (SMS) text messaging reminders to enter weight on website. Entered data were tracked and displayed graphically and in a body (BMI) silhouette.	Personalized automated enrollment reports suggesting appropriate weight-loss goals and key behavior changes required for success. Eating behaviors targeted included total energy, saturated fat and fiber intake, daily servings of fruit and vegetables, high-risk eating behaviors (eg, skipping meals, not eating breakfast, drinking soft drinks) and nonhungry eating triggers.
Individualized daily calorie targets to facilitate 0.5-1 kg weight loss per week (~2600 kJ less than their estimated energy requirements).	Weekly automated personalized feedback for key elements of diet and physical activity based on diary entries; usage patterns for website features; and level of success with weight loss. Eating behaviors targeted were consistent with the enrollment reports (Figure 2).
Access to weekly low-fat menu plan and grocery lists designed to meet nutrient reference values [16] and assigned calorie target.	Reminders to use the online diary, visit the site, and/or weigh-in. The reminder schedule included an initial reminder email; if no response, a text message; if no response, a phone call.
Web-based food and exercise diary to monitor energy intake and energy expenditure. Daily and weekly calculations of energy balance and nutrition summaries compared with recommended nutrient targets if food entries made in online diary.	
Online education in the form of weekly tutorials, fact sheets, meal, and exercise plans and weekly challenges.	
Social support via online discussion forums.	

### Outcome Measures

Participant assessments were conducted at the University of Newcastle at baseline, 12, and 24 weeks. Blinded research assistants conducted assessments for all groups, and participants were reminded at each assessment not to discuss group allocation.

Height was measured to 0.1 cm using the stretch stature method on a Harpenden portable stadiometer (Holtain Limited, Dyfed, UK). Weight was measured in light clothing, without shoes on a digital scale to 0.01 kg (CH-150kp, A&D Mercury Pty Ltd, Australia) and the primary outcome of BMI (kg/m^2^) calculated as weight (kg)/height (m)^2^. Waist circumference was measured to 0.1 cm using a nonextensible steel tape (KDSF10-02, KDS Corporation, Osaka, Japan) at 2 points: (1) level with the umbilicus and (2) at the narrowest point between the lower costal border and the umbilicus. Waist-to-height ratio was then calculated. Blood pressure and heart rate were measured using an automated blood pressure monitor (NISSEI/DS-105E digital electronic blood pressure monitor; Nihon Seimitsu Sokki Co Ltd, Gunma, Japan) under standardized conditions. Blood samples were collected with participants advised to fast overnight and analyzed for lipids (total cholesterol, low-density lipoprotein [LDL] and high-density lipoprotein [HDL], cholesterol, and triglycerides), glucose, and insulin using standard automated techniques at a single National Association of Testing Authorities accredited pathology service.

Dietary intake was assessed using the Australian Eating Survey (AES), a 120-item semiquantitative food-frequency questionnaire (FFQ). The AES has been evaluated for reliability and relative validity and demonstrates acceptable accuracy for ranking nutrient intakes in Australian adults [18]. Nutrient intakes are calculated using the Australian food composition database [19], and analyzed using a standard protocol.

The 18-item Three-Factor Eating Questionnaire-R18 (TFEQ-R18) was used to measure cognitive restraint, uncontrolled eating, and emotional eating [20]. Quality of life was assessed using the SF-36 version 2.0 (QualityMetric Incorporated, Lincoln, RI, USA), a multipurpose, generic short-form health survey consisting of an 8-scale profile of functional health and well-being scores and psychometrically based physical and mental health summary measures [21].

The International Physical Activity Questionnaire-short form (IPAQ-SF) was used to estimate total metabolic equivalent (MET)-minutes/week [22]. Pedometers were used to measure steps per day for 7 consecutive days (Yamax SW700; Yamax Corporation, Kumamoto City, Japan) with step counts adjusted for additional self-reported physical activity (eg, contact sports, swimming, cycling).

### Statistical Analysis

Continuous data were summarized using descriptive statistics including mean (SD) and categorical data as category percentages. Demographic and baseline variables were compared between treatment groups using analysis of variance (ANOVA) for continuous variables and chi-square tests for categorical variables. Analysis of covariance (ANCOVA) was used to test for differences in outcomes at 12 weeks and 24 weeks between treatment groups after adjusting for the baseline value of that outcome. The model outcome was the variable of interest at 12 or 24 weeks with the baseline level used as a covariate. The only other variable included in the model was sex. Differences and 95% confidence intervals between treatment groups in the outcome at each time point were estimated using the least squares means from the ANCOVA models.

The intention-to-treat (ITT) population includes all participants who were randomized into 1 of the 2 treatment groups. For participants who had missing data at 12 or 24 weeks, their missing data was imputed using the last observation carried forward (LOCF) and baseline observation carried forward (BOCF) approach. The completer population includes all individuals who attended the 24-week assessment, and subgroup analyses are based on this population.

An additional analysis was conducted using a generalized linear mixed model (GLMM) to test for a difference between groups across the combined 12-week and 24-week time points. The outcome in this model was the individual’s outcome at the 2 posttreatment assessments; the main predictor of interest was treatment group with the baseline value of the outcome included as a covariate. Sex was also included as a covariate in these models because it is a common confounding factor. All analyses were programmed in Stata v11 or SAS v9.2 (StataCorp LP, College Station, TX, USA).

## Results

### Baseline Characteristics

Of the 591 participants assessed for eligibility, 309 (129 males, 180 females) were initially randomized into the 3 groups (basic: n=99, enhanced: n=106, or waitlist control: n=104). After 12 weeks, the control group, of whom 8 were lost to follow-up, were rerandomized (96 participants, 52 enhanced, 44 basic) into the trial. Therefore, in the current analysis, 301 participants (125 males, 176 females) were randomized to the basic (n=143) or enhanced (n=158) groups (Figure 1).

Participants who were randomized to the basic group were similar at baseline to those randomized to the enhanced group for all demographic and other baseline characteristics (Table 2). Mean age of participants was 42 years (SD 10.2), most were overweight or obese (BMI 30 to <35), Australian born, and reported a weekly household income of ≥AU $1500.

### Retention at 12 and 24 Weeks

Participant flow through the trial (Figure 1) shows the number of participants who were randomized to each treatment condition, the number who withdrew with reasons, and the number who had data at 12 and 24 weeks. There was no significant difference in retention rates between the basic (74.7%) and enhanced (84.9%) groups after 12 weeks (*P*=.66); however, more enhanced group participants attended the 24-week assessments (basic 68.5%, enhanced 81.0%, *P*=.01).

### Changes in Weight, Body Mass Index, and Waist Circumference

Weight, BMI, and waist circumference were significantly lower than baseline at 12 and 24 weeks in each group. Change in the primary outcome of BMI was similar between those randomized to the basic and enhanced groups at 12 and 24 weeks after treatment in the LOCF (Table 3), BOCF (Table 4), and completers (Table 5) analyses. For the LOCF (basic mean -3.6, SD 4.9; enhanced mean -4.3, SD 6.4), BOCF (basic mean -3.2, SD 4.7; enhanced mean -4.2, 6.3), and completers analysis (basic mean -3.9, SD 4.1; enhanced mean -4.6, SD 4.8), there were no significant between-group differences for the mean percentage weight loss at 24 weeks or the proportion of participants achieving clinically important weight losses of ≥5% [23] at 24 weeks (LOCF: basic 31.5%, enhanced 38.0%; BOCF: basic 28.7%, enhanced 36.7%; completers: basic 41.2%, enhanced 45.7%).

### Secondary Outcomes

There was only 1 significant difference in secondary outcomes between the basic and enhanced groups in the LOCF (Table 3), BOCF (Table 4), and completers (Table 5) analyses. The BOCF analyses found that the enhanced group demonstrated a significantly greater (*P*=.03) reduction in resting heart rate than the basic group after 24 weeks.

### Subgroup Analyses

The change in primary and secondary outcomes within treatment groups was similar across all subgroups (sex, age, BMI category) of the completer population at 12 or 24 weeks (data not presented). There were no statistically significant interactions between treatment group and sex (*P*=.52), treatment group and BMI category (*P*=.45), or treatment group and age group (*P*=.72) for the outcome of weight.

### Website Usage

There was a significantly greater website usage in the enhanced group compared to the basic group at both 12 and 24 weeks with the enhanced group logging on an additional 10 days over the first 12 weeks and 12 days over 24 weeks (*P*=.002) (Table 6). A similar result was found for the completers population (*P*=.02).

In the completers population, significant correlations were found between the percentage weight loss at 12 and 24 weeks and total website usage from baseline to 12 weeks (*r* = –0.50, *P*<.001) and 24 weeks (*r* = –0.50, *P*<.001), respectively (data not shown). Participants who achieved clinically significant (≥5%) weight loss at 12 and 24 weeks used the website on significantly more days from baseline to 12 weeks (median 44 vs 13 days, *P*<.001) and 24 weeks (median 58 vs 16 days, *P*<.001) than those with <5% weight loss.

**Table 2:** Demographic and other baseline characteristics by treatment group.

Characteristic		Treatment group		Total (N=301)	*P* value^a^
		Basic (n=143)	Enhanced (n=158)		
Sex (male), n (%)		59 (47.2)	66 (52.8 )	125 (41.5 )	.93
**BMI group strata**					
	25 to <30	50 (46.7 )	57 (53.3 )	107 (35.5)	.97
	30 to <35	58 (48.3)	62 (51.7)	120 (39.9)	
	35 to <40	35 (47.3)	39 (52.7)	74 (24.6)	
Current or previous smoker (never smoked), n (%)		128 (47.8)	140 (52.2)	268 (89.0)	.91
**Highest level of education, n (%)**					
	High school	43 (47.8)	47 (52.2)	90 (29.90)	.60
	Trade/diploma	46 (44.2)	58 (55.8)	104 (34.6)	
	University degree	37 (54.4)	31 (45.60)	68 (2.6)	
	Higher university degree	17 (44.7)	21 (55.3)	38 (12.6)	
**Weekly household income (AU $), n (%)**					
	<$700	12 (50.0)	12 (50.0)	24 (8.0)	.77
	$700 to <$1000	8 (50.0)	8 (50.0)	16 (5.6)	
	$1000 to <$1400	20 (57.1)	15 (42.9)	35 (11.6)	
	≥$1500	92 (46.0)	108 (54.0)	200 (66.4)	
Country of birth (Australia), n (%)		132 (48.4)	141 (51.6)	273 (90.7)	.45
Age (years), mean (SD)		41.9 (10.1)	42.0 (10.3)	41.9 (10.2)	.91
Height (m), mean (SD)		1.7 (0.1)	1.7 (0.1)	1.7 (0.1)	.53
Weight (kg), mean (SD)		94.4 (15.5)	93.4 (13.9)	93.9 (14.7)	.53
Body mass index (kg/m^2^), mean (SD)		32.2 (3.7)	32.2 (4.1)	32.2 (3.9)	.95
Waist circumference at umbilicus (cm), mean (SD)		106.8 (10.2)	106.7 (11.5)	106.7 (10.9)	.98
Waist circumference at narrowest point (cm), mean (SD)		98.3 (11.6)	97.8 (11.2)	98.1 (11.0)	.70
Waist-to-height ratio at umbilicus, mean (SD)		0.6 (0.1)	0.6 (0.1)	0.6 (0.1)	.76
Waist-to-height ratio at narrowest point, mean (SD)		0.58 (0.06)	0.58 (0.06)	0.58 (0.06)	.94
Systolic blood pressure (mmHg), mean (SD)		121 (13)	121 (12)	121 (12)	.97
Diastolic blood pressure (mmHg), mean (SD)		79 (10)	79 (10)	79 (10)	.88
Resting heart rate (bpm), mean (SD)		69 (11)	68 (10)	68 (10)	.56
Total serum cholesterol (mmol/L), mean (SD)		5.1 (0.9)	5.1 (1.0)	5.1 (1.0)	.54
LDL cholesterol (mmol/L), mean (SD)		3.1 (0.8)	3.0 (0.9)	3.1 (0.8)	.26
HDL cholesterol (mmol/L), mean (SD)		1.3 (0.3)	1.3 (0.3)	1.3 (0.3)	.78
Triglycerides (mmol/L), mean (SD)		1.6 (0.8)	1.8 (1.1)	1.7 (1.0)	.09
LDL to HDL ratio, mean (SD)		2.53 (0.79)	2.39 (0.77)	2.46 (0.78)	.14
Glucose (mmol/L), mean (SD)		4.7 (0.7)	4.7 (0.6)	4.7 (0.7)	.35
Insulin (mIU/L), mean (SD)		11.1 (11.7)	10.5 (11.3)	10.8 (11.4)	.67
Physical functioning (SF36), mean (SD)		85.9 (13.5)	83.9 (18.4)	84.9 (16.3)	.30
Mental health (SF36), mean (SD)		73.5 (16.1)	73.2 (17.4)	73.4 (16.8)	.89
Total physical activity MET (min/week), mean (SD)		3028 (3188)	2877 (3100)	2948 (3137)	.69
Cognitive restraint scale, mean (SD)		13.3 (3.0)	13.4 (2.9)	13.3 (3.0)	.70
Uncontrolled eating scale, mean (SD)		21.1 (4.8)	20.9 (5.3)	21.0 (5.0)	.63
Emotional eating score, mean (SD)		7.7 (2.3)	7.7 (2.6)	7.7 (2.5)	.93
Total energy intake, mean (SD)		9983 (3278)	9972 (3236)	9977 (3251)	.98

^a^ *P* values are from ANOVA for continuous measures and from a chi-square tests for categorical measures.

**Table 3:** Mean change in a range of variables from baseline to 12 weeks and baseline to 24 weeks within each treatment group and the least squares mean (LSM) difference in change between treatment groups (ITT population LOCF approach).

Characteristic and follow-up time		Treatment group Mean change (SD)		Absolute difference between groups LSM (95% CI)	*P* values for group effect	
		Basic	Enhanced	Enhanced vs basic	Difference at follow-up	Difference between groups
**Weight (kg)**						
	12 weeks	–2.7 (4.0)	–3.3 (4.5)	0.6 (–0.3, 1.6)	.21	.21
	24 weeks	–3.3 (4.7)	–4.0 (6.2)	0.7 (–0.6, 2.0)	.27	
**Percentage weight loss (%)**						
	12 weeks	–2.90 (4.09)	–3.61 (4.69)	0.71 (–0.30, 1.71)	.17	.21
	24 weeks	–3.56 (4.94)	–4.28 (6.38)	0.71 (–0.59, 2.02)	.28	
**Attained 5% weight loss (%)**						
	12 weeks	24.5 (43.1)	32.9 (47.1)	8.4 (–1.9, 18.7)	.11	.13
	24 weeks	31.5 (46.6)	38.0 (48.7)	6.5 (–4.3, 17.4)	.23	
**Systolic blood pressure (mmHg)**						
	12 weeks	–3.40 (10.31)	–3.94 (9.66)	0.52 (–1.60, 2.65)	.63	.93
	24 weeks	–3.00 (10.11)	–2.33 (11.20)	0.70 (–1.46, 2.86)	.53	
**Diastolic blood pressure (mmHg)**						
	12 weeks	–1.82 (8.50)	–2.30 (7.57)	0.54 (–1.14, 2.22)	.53	.78
	24 weeks	–1.18 (8.97)	–1.03 (8.05)	0.09 (–1.68, 1.86)	.92	
**Body mass index (kg/m^2^)**						
	12 weeks	–0.9 (1.3)	–1.1 (1.5)	0.2 (–0.1, 0.5)	.28	.27
	24 weeks	–1.1 (1.5)	–1.3 (2.0)	0.2 (–0.2, 0.6)	.29	
**Resting heart rate (bpm)**						
	12 weeks	–1.33 (7.12)	–2.35 (6.49)	1.20 (–0.23, 2.64)	.10	.05
	24 weeks	–1.62 (7.69)	–3.03 (7.22)	1.59 (–0.02, 3.19)	.05	
**Waist circumference at umbilicus (cm)**						
	12 weeks	–3.4 (4.5)	–3.6 (5.3)	0.2 (–0.9, 1.3)	.73	.38
	24 weeks	–4.4 (5.3)	–5.3 (6.7)	0.9 (–0.5, 2.3)	.22	
**Waist circumference at narrowest point (cm)**						
	12 weeks	–2.5 (4.3)	–3.4 (4.8)	0.9 (–0.1, 1.9)	.09	.10
	24 weeks	–3.1 (4.6)	–4.0 (6.2)	0.9 (–0.3, 2.2)	.15	
**Waist-to-height ratio at umbilicus**						
	12 weeks	–0.02 (0.03)	–0.02 (0.03)	0.00 (–0.01, 0.01)	.86	.49
	24 weeks	–0.03 (0.03)	–0.03 (0.04)	0.00 (–0.00, 0.01)	.28	
**Waist-to-height ratio at narrowest point**						
	12 weeks	–0.01 (0.02)	–0.02 (0.03)	0.00 (–0.00, 0.01)	.11	.14
	24 weeks	–0.02 (0.03)	–0.02 (0.04)	0.00 (–0.00, 0.01)	.21	
**Total serum cholesterol (mmol/L)**						
	12 weeks	–0.08 (0.50)	–0.19 (0.58)	0.12 (–0.00, 0.24)	.06	.06
	24 weeks	0.01 (0.52)	–0.08 (0.62)	0.09 (–0.03, 0.22)	.15	
**LDL cholesterol (mmol/L)**						
	12 weeks	–0.00 (0.45)	–0.07 (0.46)	0.09 (–0.02, 0.19)	.11	.13
	24 weeks	0.04 (0.49)	–0.01 (0.50)	0.07 (–0.05, 0.18)	.27	
**HDL cholesterol (mmol/L)**						
	12 weeks	–0.00 (0.14)	–0.01 (0.16)	0.00 (–0.03, 0.04)	.93	.92
	24 weeks	0.02 (0.13)	0.02 (0.17)	0.00 (–0.03, 0.04)	.80	
**Triglycerides (mmol/L)**						
	12 weeks	–0.16 (0.63)	–0.26 (0.66)	0.05 (–0.08, 0.18)	.48	.30
	24 weeks	–0.09 (0.64)	–0.23 (0.66)	0.09 (–0.04, 0.23)	.18	
**LDL to HDL ratio**						
	12 weeks	0.00 (0.40)	–0.04 (0.35)	0.06 (–0.03, 0.15)	.21	.21
	24 weeks	–0.01 (0.42)	–0.04 (0.37)	0.04 (–0.05, 0.14)	.35	
**Glucose (mmol/L)**						
	12 weeks	–0.22 (0.55)	–0.22 (0.55)	0.02 (–0.10, 0.14)	.78	.37
	24 weeks	–0.07 (0.46)	–0.12 (0.54)	0.08 (–0.03, 0.18)	.17	
**Insulin (mIU/L)**						
	12 weeks	–1.76 (10.91)	–1.80 (5.47)	0.29 (–1.25, 1.82)	.71	.81
	24 weeks	–2.51 (10.36)	–2.31 (6.18)	0.04 (–1.47, 1.55)	.96	
**Physical functioning (SF36)**						
	12 weeks	2.48 (20.05)	4.71 (15.74)	1.14 (–2.34, 4.62)	.52	.63
	24 weeks	3.58 (10.89)	4.62 (15.03)	0.17 (–2.30, 2.64)	.89	
**Mental health (SF36)**						
	12 weeks	2.13 (15.06)	4.04 (13.43)	1.58 (–1.41, 4.57)	.30	.31
	24 weeks	1.77 (14.83)	3.82 (23.80)	1.68 (–2.69, 6.04)	.45	
**Total physical activity MET (min/week)**						
	12 weeks	215.29 (2448.3)	373.56 (2467.1)	96.19 (–424.3, 616.66)	.72	.36
	24 weeks	203.58 (2668.5)	619.84 (3156.7)	358.58 (–285.1, 1002.3)	.27	
**Average step count per day**						
	12 weeks	319.92 (2481.3)	1059.8 (3094.2)	675.22 (–11.32, 1361.8)	.05	.06
	24 weeks	94.15 (2303.2)	706.99 (3173.9)	548.38 (–142.9, 1239.6)	.12	
**Cognitive restraint scale**						
	12 weeks	1.32 (2.72)	1.74 (3.28)	0.48 (–0.15, 1.11)	.14	.08
	24 weeks	1.36 (2.96)	1.90 (3.37)	0.59 (–0.09, 1.27)	.09	
**Uncontrolled eating scale**						
	12 weeks	–1.71 (3.44)	–1.78 (3.41)	0.14 (–0.58, 0.86)	.70	.65
	24 weeks	–1.67 (3.59)	–1.76 (3.62)	0.17 (–0.59, 0.93)	.66	
**Emotional eating score**						
	12 weeks	–0.37 (1.38)	–0.48 (1.56)	0.10 (–0.22, 0.41)	.54	.43
	24 weeks	–0.45 (1.68)	–0.61 (1.72)	0.15 (–0.21, 0.51)	.41	
**Total energy intake**						
	12 weeks	–985.5 (2386.7)	–1248 (2458.1)	267.74 (–186.4, 721.90)	.25	.57
	24 weeks	–952.4 (2293.8)	–929.7 (2363.6)	18.23 (–439.2, 475.66)	.94	

**Table 4:** Mean change in a range of variables from baseline to 12 weeks and baseline to 24 weeks within each treatment group and the least squares mean (LSM) difference in change between treatment groups (ITT population BOCF approach).

Characteristic and follow-up time		Treatment group Mean change (SD)		Absolute difference between groups LSM (95% CI)	*P* values for group effect	
		Basic	Enhanced	Enhanced vs basic	Difference at follow-up	Difference between groups
**Weight (kg)**						
	12 weeks	–2.7 (4.0)	–3.3 (4.5)	0.6 (–0.3, 1.6)	.21	.13
	24 weeks	–3.0 (4.5)	–3.9 (6.2)	1.0 (–0.2, 2.2)	.11	
**Percentage weight loss (%)**						
	12 weeks	–2.90 (4.09)	–3.61 (4.69)	0.71 (–0.30, 1.71)	.17	.12
	24 weeks	–3.17 (4.74)	–4.19 (6.34)	1.02 (–0.27, 2.30)	.12	
**Attained 5% weight loss (%)**						
	12 weeks	24.5 (43.1)	32.9 (47.1)	8.43 (–1.87, 18.73)	.11	.09
	24 weeks	28.7 (45.4)	36.7 (48.4)	8.07 (–2.58, 18.73)	.13	
**Systolic blood pressure (mmHg)**						
	12 weeks	–3.40 (10.31)	–3.94 (9.66)	0.52 (–1.60, 2.65)	.63	.92
	24 weeks	–2.46 (9.71)	–2.14 (10.85)	0.35 (–1.77, 2.47)	.75	
**Diastolic blood pressure (mmHg)**						
	12 weeks	–1.82 (8.50)	–2.30 (7.57)	0.54 (–1.14, 2.22)	.53	.68
	24 weeks	–0.89 (8.59)	–0.91 (7.60)	0.08 (–1.62, 1.78)	.93	
**Body mass index (kg/m^2^)**						
	12 weeks	–0.93 (1.31)	–1.11 (1.52)	0.18 (–0.14, 0.50)	.28	.20
	24 weeks	–0.97 (1.45)	–1.24 (2.00)	0.28 (–0.12, 0.68)	.17	
**Resting heart rate (bpm)**						
	12 weeks	–1.33 (7.12)	–2.35 (6.49)	1.20 (–0.23, 2.64)	.10	.03
	24 weeks	–1.30 (7.15)	–2.78 (6.86)	1.64 (0.12, 3.15)	.03	
**Waist circumference at umbilicus (cm)**						
	12 weeks	–3.37 (4.52)	–3.57 (5.31)	0.20 (–0.93, 1.33)	.73	.26
	24 weeks	–3.81 (5.17)	–4.93 (6.73)	1.12 (–0.25, 2.48)	.11	
**Waist circumference at narrowest point (cm)**						
	12 weeks	–2.48 (4.27)	–3.37 (4.80)	0.90 (–0.13, 1.92)	.09	.06
	24 weeks	–2.73 (4.26)	–3.79 (6.19)	1.08 (–0.13, 2.29)	.08	
**Waist-to-height ratio at umbilicus**						
	12 weeks	–0.02 (0.03)	–0.02 (0.03)	0.00 (–0.01, 0.01)	.86	.35
	24 weeks	–0.02 (0.03)	–0.03 (0.04)	0.01 (–0.00, 0.01)	.15	
**Waist-to-height ratio at narrowest point**						
	12 weeks	–0.01 (0.02)	–0.02 (0.03)	0.00 (–0.00, 0.01)	.11	.09
	24 weeks	–0.02 (0.02)	–0.02 (0.04)	0.01 (–0.00, 0.01)	.12	
**Total serum cholesterol (mmol/L)**						
	12 weeks	–0.08 (0.50)	–0.19 (0.58)	0.12 (–0.00, 0.24)	.06	.06
	24 weeks	0.01 (0.44)	–0.06 (0.58)	0.08 (–0.04, 0.19)	.20	
**LDL cholesterol (mmol/L)**						
	12 weeks	–0.00 (0.45)	–0.07 (0.46)	0.09 (–0.02, 0.19)	.11	.10
	24 weeks	0.02 (0.39)	–0.02 (0.46)	0.06 (–0.04, 0.16)	.27	
**HDL cholesterol (mmol/L)**						
	12 weeks	–0.00 (0.14)	–0.01 (0.16)	0.00 (–0.03, 0.04)	.93	.86
	24 weeks	0.02 (0.10)	0.02 (0.16)	0.01 (–0.02, 0.04)	.68	
**Triglycerides (mmol/L)**						
	12 weeks	–0.16 (0.63)	–0.26 (0.66)	0.05 (–0.08, 0.18)	.48	.27
	24 weeks	–0.08 (0.43)	–0.21 (0.65)	0.08 (–0.04, 0.19)	.18	
**LDL to HDL ratio**						
	12 weeks	0.00 (0.40)	–0.04 (0.35)	0.06 (–0.03, 0.15)	.21	.23
	24 weeks	–0.02 (0.34)	–0.04 (0.36)	0.03 (–0.06, 0.11)	.51	
**Glucose (mmol/L)**						
	12 weeks	–0.22 (0.55)	–0.22 (0.55)	0.02 (–0.10, 0.14)	.78	.22
	24 weeks	–0.02 (0.38)	–0.10 (0.50)	0.10 (0.00, 0.20)	.05	
**Insulin (mIU/L)**						
	12 weeks	–1.76 (10.91)	–1.80 (5.47)	0.29 (–1.25, 1.82)	.71	.82
	24 weeks	–2.37 (9.85)	–2.16 (5.92)	0.02 (–1.43, 1.47)	.98	
**Physical functioning (SF36)**						
	12 weeks	2.48 (20.05)	4.71 (15.74)	1.14 (–2.34, 4.62)	.52	.90
	24 weeks	3.48 (10.40)	3.38 (13.63)	0.81 (–1.58, 3.21)	.50	
**Mental health (SF36)**						
	12 weeks	2.13 (15.06)	4.04 (13.43)	1.58 (–1.41, 4.57)	.30	.22
	24 weeks	0.82 (11.74)	3.15 (23.39)	2.03 (–2.11, 6.17)	.34	
**Total physical activity MET (min/week)**						
	12 weeks	215.29 (2448.3)	373.56 (2467.1)	96.19 (–424.3, 616.66)	.72	.20
	24 weeks	136.47 (2303.8)	653.82 (2882.0)	474.17 (–112.4, 1060.7)	.11	
**Average step count per day**						
	12 weeks	319.92 (2481.3)	1059.8 (3094.2)	675.22 (–11.32, 1361.8)	.05	.05
	24 weeks	36.69 (1838.9)	485.19 (2766.5)	405.53 (–191.5, 1002.6)	.18	
**Cognitive restraint scale**						
	12 weeks	1.32 (2.72)	1.74 (3.28)	0.48 (–0.15, 1.11)	.14	.12
	24 weeks	1.32 (2.75)	1.71 (3.10)	0.43 (–0.22, 1.08)	.19	
**Uncontrolled eating scale**						
	12 weeks	–1.71 (3.44)	–1.78 (3.41)	0.14 (–0.58, 0.86)	.70	.73
	24 weeks	–1.55 (3.37)	–1.56 (3.48)	0.09 (–0.65, 0.82)	.82	
**Emotional eating score**						
	12 weeks	–0.37 (1.38)	–0.48 (1.56)	0.10 (–0.22, 0.41)	.54	.38
	24 weeks	–0.40 (1.59)	–0.57 (1.68)	0.17 (–0.19, 0.52)	.36	
**Total energy intake**						
	12 weeks	–985.5 (2386.7)	–1248 (2458.1)	267.74 (–186.4, 721.90)	.25	.54
	24 weeks	–787.1 (2095.4)	–761.4 (2046.2)	22.36 (–407.4, 452.10)	.92	

**Table 5:** Mean change in a range of variables from baseline to 12 weeks and baseline to 24 weeks within each treatment group and the least squares mean (LSM) difference in change between treatment groups (completers population).

Characteristic and follow-up time		Treatment group Mean change (SD)		Absolute difference between groups LSM (95% CI)	*P* values for group effect	
		Basic	Enhanced	Enhanced vs basic	Difference at follow-up	Difference between groups
**Weight (kg)**						
	12 weeks	–3.7 (4.0)	–4.3 (4.6)	0.6 (–0.6, 1.8)	.34	.35
	24 weeks	–4.1 (4.7)	–4.8 (6.6)	0.7 (–0.8, 2.3)	.35	
**Percentage weight loss (%)**						
	12 weeks	–3.89 (4.10)	–4.59 (4.81)	0.69 (–0.54, 1.92)	.27	.27
	24 weeks	–4.48 (4.91)	–5.18 (6.70)	0.71 (–0.88, 2.31)	.38	
**Attained 5% weight loss (%)**						
	12 weeks	33.7 (47.5)	41.3 (49.4)	7.65 (–5.65, 20.94)	.26	.31
	24 weeks	41.2 (49.5)	45.7 (50.0)	4.76 (–8.40, 17.92)	.48	
**Systolic blood pressure (mmHg)**						
	12 weeks	–4.91 (12.38)	–4.91 (10.63)	0.37 (–2.48, 3.21)	.80	.65
	24 weeks	–3.98 (12.11)	–2.95 (12.65)	0.78 (–2.15, 3.71)	.60	
**Diastolic blood pressure (mmHg)**						
	12 weeks	–2.87 (10.33)	–2.86 (8.37)	0.43 (–1.72, 2.59)	.69	.91
	24 weeks	–1.43 (10.89)	–1.25 (8.90)	0.22 (–2.14, 2.58)	.85	
**Body mass index (kg/m^2^)**						
	12 weeks	–1.27 (1.33)	–1.42 (1.57)	0.15 (–0.26, 0.55)	.48	.48
	24 weeks	–1.53 (1.57)	–1.70 (2.17)	0.16 (–0.37, 0.70)	.55	
**Resting heart rate (bpm)**						
	12 weeks	–1.90 (8.31)	–3.07 (7.18)	1.37 (–0.48, 3.21)	.15	.05
	24 weeks	–2.09 (8.97)	–3.81 (7.78)	1.76 (–0.34, 3.87)	.10	
**Waist circumference at umbilicus (cm)**						
	12 weeks	–4.44 (4.56)	–4.51 (5.50)	0.02 (–1.38, 1.42)	.98	.64
	24 weeks	–6.13 (5.36)	–6.83 (7.06)	0.56 (–1.16, 2.29)	.52	
**Waist circumference at narrowest point (cm)**						
	12 weeks	–3.18 (4.54)	–4.28 (5.01)	1.05 (–0.24, 2.34)	.11	.15
	24 weeks	–4.39 (4.68)	–5.25 (6.75)	0.82 (–0.81, 2.44)	.32	
**Waist-to-height ratio at umbilicus**						
	12 weeks	–0.03 (0.03)	–0.03 (0.03)	0.00 (–0.01, 0.01)	.91	.75
	24 weeks	–0.04 (0.03)	–0.04 (0.04)	0.00 (–0.01, 0.01)	.65	
**Waist-to-height ratio at narrowest point**						
	12 weeks	–0.02 (0.03)	–0.02 (0.03)	0.01 (–0.00, 0.01)	.14	.19
	24 weeks	–0.03 (0.03)	–0.03 (0.04)	0.00 (–0.01, 0.01)	.42	
**Total serum cholesterol (mmol/L)**						
	12 weeks	–0.12 (0.54)	–0.23 (0.65)	0.12 (–0.04, 0.28)	.14	.09
	24 weeks	0.02 (0.57)	–0.09 (0.70)	0.12 (–0.06, 0.30)	.20	
**LDL cholesterol (mmol/L)**						
	12 weeks	–0.02 (0.45)	–0.08 (0.53)	0.08 (–0.06, 0.22)	.27	.26
	24 weeks	0.04 (0.52)	–0.02 (0.57)	0.09 (–0.07, 0.26)	.27	
**HDL cholesterol (mmol/L)**						
	12 weeks	–0.01 (0.14)	–0.01 (0.18)	0.01 (–0.04, 0.05)	.76	.71
	24 weeks	0.03 (0.13)	0.04 (0.19)	0.00 (–0.04, 0.05)	.86	
**Triglycerides (mmol/L)**						
	12 weeks	–0.21 (0.64)	–0.34 (0.73)	0.06 (–0.10, 0.22)	.47	.21
	24 weeks	–0.14 (0.56)	–0.30 (0.77)	0.07 (–0.09, 0.24)	.38	
**LDL to HDL ratio**						
	12 weeks	–0.02 (0.44)	–0.05 (0.40)	0.05 (–0.07, 0.18)	.38	.43
	24 weeks	–0.04 (0.46)	–0.07 (0.44)	0.04 (–0.10, 0.17)	.61	
**Glucose (mmol/L)**						
	12 weeks	–0.31 (0.64)	–0.27 (0.61)	0.00 (–0.16, 0.16)	.99	.35
	24 weeks	–0.03 (0.50)	–0.15 (0.61)	0.15 (–0.00, 0.29)	.05	
**Insulin (mIU/L)**						
	12 weeks	–2.69 (13.26)	–2.25 (6.14)	0.48 (–1.13, 2.08)	.56	.61
	24 weeks	–4.03 (12.62)	–3.15 (6.95)	0.20 (–1.34, 1.73)	.80	
**Physical functioning (SF36)**						
	12 weeks	3.54 (24.91)	5.21 (16.40)	0.57 (–3.93, 5.08)	.80	.88
	24 weeks	5.57 (12.74)	4.78 (15.93)	1.25 (–1.89, 4.40)	.43	
**Mental health (SF36)**						
	12 weeks	2.36 (18.09)	4.88 (14.97)	1.39 (–2.51, 5.28)	.48	.53
	24 weeks	1.31 (14.87)	4.38 (27.63)	1.87 (–4.18, 7.93)	.54	
**Total physical activity MET (min/week)**						
	12 weeks	164.57 (2799.8)	432.99 (2735.8)	214.87 (–403.0, 832.76)	.49	.28
	24 weeks	230.41 (2997.8)	932.18 (3408.2)	491.04 (–381.0, 1363.0)	.27	
**Average step count per day**						
	12 weeks	418.22 (3102.2)	1598.7 (3600.4)	1139.3 (118.76, 2159.9)	.03	.06
	24 weeks	91.73 (2926.8)	808.65 (3543.6)	587.05 (–604.7, 1778.8)	.33	
**Cognitive restraint scale**						
	12 weeks	2.11 (2.94)	2.29 (3.48)	0.01 (–0.78, 0.79)	.98	.66
	24 weeks	2.13 (3.24)	2.44 (3.46)	0.19 (–0.69, 1.07)	.66	
**Uncontrolled eating scale**						
	12 weeks	–2.60 (3.82)	–2.26 (3.76)	0.12 (–0.81, 1.05)	.81	.96
	24 weeks	–2.46 (3.98)	–2.20 (3.96)	0.07 (–0.92, 1.07)	.89	
**Emotional eating score**						
	12 weeks	–0.53 (1.61)	–0.59 (1.75)	0.06 (–0.37, 0.48)	.79	.58
	24 weeks	–0.63 (1.98)	–0.79 (1.94)	0.14 (–0.36, 0.64)	.58	
**Total energy intake**						
	12 weeks	–1480 (2695.4)	–1467 (2428.2)	90.91 (–462.8, 644.63)	.75	.82
	24 weeks	–1285 (2559.6)	–1047 (2338.7)	208.94 (–379.6, 797.48)	.48	

**Table 6:** Mean change in total website usage for the completers population and the ITT with LOCF from baseline to 12 weeks and baseline to 24 weeks within each treatment group.

Characteristic and follow-up time			Treatment group Mean change (SD)		Absolute difference between groups LSM (95% CI)	*P* values for group effect	
			Basic	Enhanced	Enhanced vs basic	Difference at follow-up	Difference between groups
**Intention-to-treat**							
	**Total website was usage since baseline (days)**						
		12 weeks	24.6 (25.5)	34.1 (28.1)	9.45 (3.34, 15.56)	.002	.002
		24 weeks	31.8 (33.9)	43.1 (34.0)	12.47 (4.73, 20.20)	.002	
**Completers**							
	**Total website was usage since baseline (days)**						
		12 weeks	29.7 (26.6)	38.7 (28.5)	9.2 (–1.9, 16.5)	.01	.02
		24 weeks	42.0 (36.1)	49.8 (33.3)	8.1 (–3.3, 19.4)	.16	

## Discussion

### Principal Findings

The aim of the study was to evaluate whether overweight and obese adults randomized to a commercial Web-based weight-loss program providing greater social support and more personalized feedback achieved a greater reduction in BMI and increased usage of program features compared to those randomized to a standard version of the program. We found no differences in weight loss or most of the secondary health outcomes between the basic and enhanced features versions of the Web-based weight-loss program after 24 weeks, despite previous reports that provision of enhanced features within Web-based formats does enhance weight-loss outcomes [5]. Mean weight loss in the current study ranged from 2 to 3 kg after 12 weeks and 3 to 4 kg after 24 weeks across both intervention groups. Both the magnitude of weight loss, and the continuance of weight loss from 12 to 24 weeks highlights that both versions are promising at the population level. The results also compare favorably to previous Web-based studies. Only 4 of 7 studies were deemed effective with a mean weight loss ≥5% [24-28]. When we examined those who achieved ≥5% weight loss, success was strongly associated with website usage, indicating that strategies to improve website usage may be beneficial to weight loss outcomes. In this regard, some aspects of the enhanced program features may be valuable because the enhanced group had a significantly lower dropout rate and greater participant engagement.

In the current study, the basic and enhanced versions may have produced similar weight loss because several of these components were similar (self-monitoring, social support, structured program) or absent (eg, counselor feedback) in both versions. Khaylis et al [29] reviewed technology-based weight-loss intervention studies and identified 5 factors that may contribute to successful weight loss: use of a structured program, self-monitoring, social support, use of an individually tailored program, and counselor feedback and communication. Although semipersonalized system-generated feedback and an escalating reminder scale to begin was provided in the enhanced group, the report may not have been specific enough to help them further improve their dietary intake, physical activity, and log-ins. The contact may have been viewed as too much contact and, therefore, contributed to nonusage. In the current study, the basic version of the Web-based weight-loss program proved effective, supported by the 0.9 kg/m^2^ reduction in BMI (2.7 kg) at 12 weeks and the 1.1 kg/m^2^ BMI reduction (3.3 kg) after 24 weeks. This degree of weight loss is similar to that in the enhanced arm of older Web-based trials. For example, in 2001, Tate et al [28] reported 3- and 6-month weight losses of -3.2 kg and -2.9 kg, respectively, in 46 adults in the enhanced arm of an Internet weight-loss trial compared to -1.0 kg and -1.3 kg for the basic group, whereas in 2006, Rothert et al [8] reported that for 1475 adults participating in the tailored (enhanced) feedback arm, the mean weight losses at 3 and 6 months were 0.8% and 0.9% body weight compared to -0.4% in the basic information-only Internet program at both 3 and 6 months. A recent 2010 study by Wing et al [6] did report significantly greater weight loss in an enhanced Web program (-3.1 kg) compared to a basic version (-1.2 kg), with mean weight loss for the enhanced similar to the basic program in the current study. Another study by Webber et al [10] reported greater weight loss using Web-based programs than the current study. Although the weight loss between a basic (minimal contact) and enhanced version of an Internet program was not significantly different, both groups achieved substantial weight losses of 5.2 kg (minimal) and 3.7 kg (enhanced) after 16 weeks [10]. However, it is difficult to compare and discern how different Web-based features may influence outcomes. Having standardized ways of describing or reporting enhanced program features would assist in making comparisons across studies and, over time, could help with identifying the set of program components that may optimize weight loss using Web-based programs. Future programs may need to segment the target population to improve feedback tailoring to specific user groups as a strategy to avoid website discontinuity, particularly in relation to some age, sex, or BMI subgroups [30]. Some groups may not need this more extensive feedback and it would be useful to identify who they are. Although there were some minor differences in outcomes across categories of age, BMI, and sex, a Web-based program may potentially benefit specific groups of program users [31,32].

In the completers population of the current study, significant correlations were found between total website usage and the percentage weight loss at 12 and 24 weeks. Participants who achieved clinically important weight loss (≥5%) at either time point used the website almost 4 times more than those who were not successful (<5% weight loss). Further, the website log-ins among those deemed successful was substantially greater than the number of log-ins reported for the enhanced group. Those with successful weight loss logged in 2 to 3 times per week, compared to just once or twice a week for those randomized to the enhanced group and less than once a week for those randomized to the basic group.

The correlation between number of log-ins and weight loss was moderate across all study participants and there was also no between-group difference. This suggests that although being allocated to the enhanced program did facilitate more frequent website log-ins, provision of additional features is not enough to facilitate greater engagement and weight loss. Further research examining which combination of website features optimize program use and reduce attrition are needed. Based on the current study, future modifications to the enhanced program would need to achieve a 50% increase in the number of participant log-ins than that in the current study. This would mean getting participants to use the program at least 2 to 3 times per week as a strategy to facilitate clinically important ≥5% weight loss. Establishing and testing these targets could ease the burden and fatigue associated with program usage targets that are not achievable or sustainable. Although between-groups differences might typically be explained by confounders such as energy intake and physical activity, these were not different between groups. It is more likely that differential use of social support features, including blogs, forums, and chat rooms, explain the between-group difference and this requires further research. We cannot tell whether the reminders schedule to log in and use of program features in the current study was the key driver of this and this also needs to be examined in future studies.

### Limitations

A limitation of the current study is that it did not have a waitlist control group at 6 months. However, this was not required to answer the research question. Attrition reduced the power to detect significant differences between groups, particularly for the secondary outcomes; however, there were a few trends suggesting that better retention would not have changed the outcomes in any substantial way. The strengths of this study include the use of an RCT, large sample size, use of blinded assessors, and the comparison of the effectiveness of the 2 versions of the weight-loss programs up to 24 weeks. Further, few commercial Web-based programs have been subjected to evaluation by RCT, with none previously conducted in Australia. Importantly, this study has demonstrated the efficacy of a commercial Web-based weight-loss program in achieving clinically important weight loss.

### Conclusions

In conclusion, commercial Web-based weight-loss programs can be effective at achieving clinically meaningful weight loss up to 24 weeks. Although adding enhanced features that provide additional feedback, reminders, and social support promotes greater retention and engagement, it does not necessarily increase weight loss substantially. Further research into Web-based features that optimize website usage, program engagement, and weight-loss success is warranted.

## Multimedia Appendix 1

CONSORT-eHEALTH checklist V1.6.2 [15].

## Multimedia Appendix 2

Sample weekly report_5.

## Multimedia Appendix 3

Sample weekly report_5a.

## Abbreviations

AES: Australian Eating Survey
ANOVA: analysis of variance
ANZCTR: Australian New Zealand Clinical Trials Registry
BMI: body mass index
BOCF: baseline observation carried forward
CONSORT: Consolidated Standards of Reporting Trials
FFQ: food-frequency questionnaire
GLMM: generalized linear mixed model
HDL: high-density lipoprotein
IPAQ-SF: International Physical Activity Questionnaire-short form
ITT: intention-to-treat
LDL: low-density lipoprotein
LOCF: last observation carried forward
LSM: least squares mean
MET: total metabolic equivalent
RCT: randomized controlled trial
SMS: short message service
TFEQ-R18: Three-Factor Eating Questionnaire-R18
