# Supplementary material for: Efficacy of Standard Versus Enhanced Features in a Web-Based Commercial Weight-Loss Program for Obese Adults, Part 2: Randomized Controlled Trial
Source: J Med Internet Res. 2013 Jul 22;15(7):e140. doi: 10.2196/jmir.2626 (PMC3786000; doi:10.2196/jmir.2626)
Supplement: Supplementary file 1 [file jmir_v15i7e140_app1.pdf]

CONSORT-EHEALTH Checklist V1.6.2 Report

(based on CONSORT-EHEALTH V1.6), available at [http://tinyurl.com/consort-ehealth-v1-6].

2626

Date completed

3/18/2013 21:54:15

by

Clare Collins

Efficacy of standard versus enhanced features web-based commercial weight loss program in obese adults: A randomized controlled trial

TITLE

1a-i) Identify the mode of delivery in the title

Efficacy of standard versus enhanced features web-based commercial weight loss program in obese adults: A randomized controlled trial

1a-ii) Non-web-based components or important co-interventions in title

"standard versus enhanced features web-based commercial"

1a-iii) Primary condition or target group in the title

Efficacy of standard versus enhanced features web-based commercial weight loss program in obese adults: A randomized controlled trial

ABSTRACT

1b-i) Key features/functionalities/components of the intervention and comparator in the METHODS section of the ABSTRACT

"An assessor-blinded RCT comparing 301 adults (42% male, 42(10) years, BMI 32.2±3.9kg/m2) who were recruited and enrolled offline, and randomly allocated to Basic or Enhanced versions of a commercially available web-based weight loss program for 24 weeks."

1b-ii) Level of human involvement in the METHODS section of the ABSTRACT

"An assessor-blinded RCT comparing 301 adults (42% male, 42(10) years, BMI 32.2±3.9kg/m2) who were recruited and enrolled offline, and randomly allocated to Basic or Enhanced versions of a commercially available web-based weight loss program for 24 weeks."

1b-iii) Open vs. closed, web-based (self-assessment) vs. face-to-face assessments in the METHODS section of the ABSTRACT

"An assessor-blinded RCT comparing 301 adults (42% male, 42(10) years, BMI 32.2±3.9kg/m2) who were recruited and enrolled offline"

1b-iv) RESULTS section in abstract must contain use data

"Retention at 24 weeks was greater in the Enhanced group versus the Basic group (B 69%, E 81%, p=0.012)."

"The Enhanced group logged in more often at both 12- and 24-weeks respectively (E 34.1 28.1 and 43.134.0 vs. B 24.6 25.5 and 31.8 33.9, p<0.002)."

1b-v) CONCLUSIONS/DISCUSSION in abstract for negative trials

"The addition of personalized e-feedback in the enhanced program provided limited additional benefits compared to a standard commercial web-based weight loss program (p<0.05). However, it does support a greater retention in the program and greater usage, which was related to weight loss."

INTRODUCTION

2a-i) Problem and the type of system/solution

"Internationally, obesity rates in adults continue to rise unabated.[1]....Web-based weight loss programs are an increasingly viable option as the majority of US and Australian households (66%[2], 72%[3] respectively) have access to broadband internet, and many adults (61% in the US) seek information on health, nutrition, and weight loss from the Internet,[4]."

"Therefore this study aimed to determine whether overweight and obese adults randomized to a commercial web-based weight loss program providing greater social support and more personalized feedback resulted in a larger reduction in BMI and usage of program features compared to those randomized to a standard version of the online program, without these features."

2a-ii) Scientific background, rationale: What is known about the (type of) system

"A meta-analysis of three web-based weight loss RCTs that compared online education-only programs with online programs that included enhanced features such as counselling, automated or therapist feedback, behavioural lessons, self-monitoring, and a bulletin board, found weight loss was increased by 2.2 kg over a 6- to 12-month period.[6] These results are supported by three other RCTs which found that the addition of online lessons with daily self-monitoring of weight, eating and exercise and computer generated feedback,[7] or the addition of peer support,[8] or individually tailored action plans [9], resulted in greater weight loss after 24 weeks[7], a trend towards a greater effect size after 12 weeks[8] and greater weight loss [9] compared to an online program without the enhanced features."

METHODS

3a) CONSORT: Description of trial design (such as parallel, factorial) including allocation ratio

"Therefore this study aimed to determine whether overweight and obese adults randomized to a commercial web-based weight loss program providing greater social support and more personalized feedback resulted in a larger reduction in BMI and usage of program features compared to those randomized to a standard version of the online program, without these features."

3b) CONSORT: Important changes to methods after trial commencement (such as eligibility criteria), with reasons

There was no change to the methods after trial commencement.

3b-i) Bug fixes, Downtimes, Content Changes

There were no changes made to the ehealth system after trial commencement.

4a) CONSORT: Eligibility criteria for participants

"Eligibility criteria included: age 18 to 60y, BMI 25 to 40 kg/m2, not participating in other weight loss programs, pass a health screen,[12] available for in-person assessments, and access to a computer with e-mail and Internet services.[11]"

4a-i) Computer / Internet literacy

No. This computer literacy was not an inclusion criterion. Only access to internet and computer.

4a-ii) Open vs. closed, web-based vs. face-to-face assessments:

"Briefly, overweight and obese adults from the Hunter community in NSW, Australia were recruited and enrolled offline in 2009."

"After baseline assessments were completed, participants were stratified by sex and BMI category (25 to <30; ≥30 to <35 or ≥35 to 40 km.m-2) and randomized using a stratified block design to either the standard (Basic, B) web-based weight loss program or the same program with additional features (Enhanced, E) (Figure 1)."

"Participant assessments were conducted at the University of Newcastle at baseline, 12 and 24 weeks.[11]"

4a-iii) Information giving during recruitment

"Written informed consent was obtained from all participants, and ethical approval obtained from the University of Newcastle Human Ethics Research Committee."

4b) CONSORT: Settings and locations where the data were collected

"Eligibility criteria included: age 18 to 60y, BMI 25 to 40 kg/m2, not participating in other weight loss programs, pass a health screen,[12] available for in-person assessments, and access to a computer with e-mail and Internet services.[11]"

"Participant assessments were conducted at the University of Newcastle at baseline, 12 and 24 weeks.[11]"

4b-i) Report if outcomes were (self-)assessed through online questionnaires

Study outcomes were assessed in person at the university. However, as part of the study design, participants were encouraged to self-report weight and dietary outcomes online.

"Participants were encouraged to self-monitor by reporting their weight or other body measurements via the website or short message service (SMS) once per week and could view graphs and charts to track their progress overtime. They were also encouraged to self-monitor their dietary intake and exercise using an online diary at least four days per week."

4b-ii) Report how institutional affiliations are displayed

Affiliations with the University of Newcastle were displayed by logo and location in the information and consent form.

"Participants were provided with free access to the Basic or Enhanced version of a commercial web-based program provided by SP Health Co Pty Ltd in Australia under the name The Biggest Loser Club."

5) CONSORT: Describe the interventions for each group with sufficient details to allow replication, including how and when they were actually administered

5-i) Mention names, credential, affiliations of the developers, sponsors, and owners

CONFLICTS OF INTEREST

"This trial was funded by an Australian Research Council Linkage Project grant (2009–2012) (LP0990414, G0189752), with SP Health as the Industry Linkage Partner Organization (G0189753).

CEC has been a nutrition consultant to SP Health Co. M Hutchesson (nee Neve) was funded by a Penn Health post-doctoral fellowship. All other authors declare that they have no competing interests."

5-ii) Describe the history/development process

This study was based on information from previous studies that have demonstrated that web-based interventions are effective in achieving weight loss. Furthermore, previous studies demonstrated that adidtional, more personalised features of web-based intervnetions increase the weight loss outcomes compared to a standard web-based apporach.

5-iii) Revisions and updating

The study had no interruptions or changes to the intervention after trial commencement.

5-iv) Quality assurance methods

The in person assessments were conducted by research personnal who were blinded to the group allocations. In addition, the study was not to be discussed during these assessments. Objective measurements of outcomes were used.

5-v) Ensure replicability by publishing the source code, and/or providing screenshots/screen-capture video, and/or providing flowcharts of the algorithms used

The web-based program used was a commercially available program. Screenshots were taken by research personnel and are available on request.

"Participants were provided with free access to the Basic or Enhanced version of a commercial web-based program provided by SP Health Co Pty Ltd in Australia under the name The Biggest Loser Club. The Basic program was the version commercially available at the time of the study (2009-10). Program features are reported in Table 1."

5-vi) Digital preservation

The following information was provided, however snapshots are available on request.

"Participants were provided with free access to the Basic or Enhanced version of a commercial web-based program provided by SP Health Co Pty Ltd in Australia under the name The Biggest Loser Club. The Basic program was the version commercially available at the time of the study (2009-10). Program features are reported in Table 1."

5-vii) Access

"Participants were provided with free access to the Basic or Enhanced version of a commercial web-based program provided by SP Health Co Pty Ltd in Australia under the name The Biggest Loser Club. The Basic program was the version commercially available at the time of the study (2009-10). Program features are reported in Table 1. The Enhanced program contained additional features to the Basic program and was provided in a closed test environment. At baseline participants were given instructions to log in and set up their program details. They were also given a company phone number in case they experienced any difficulties in logging in. Participants did not receive any training on program use in order to mirror the commercial program engagement experience and increase external validity. Participants were blinded to group allocation, and accessed the website using their usual Internet connection."

"Online information was provided weekly (calorie-controlled, low-fat menu plans and grocery lists; physical activity plan based on exercise preferences; educational tips and challenges) which participants were prompted to access via a weekly email newsletter."

5-viii) Mode of delivery, features/functionalities/components of the intervention and comparator, and the theoretical framework

"The 12 week web-based programs were based on social cognitive theory[13] and have been described elsewhere in detail [11, 14]. Key behavior change mediators targeted included self-efficacy, goal setting, self-monitoring, outcome expectations, and social support. An individualized daily energy intake target to facilitate a weight loss of 0.5 kg to 1 kg per week was set, as well as a goal weight. Participants were encouraged to self-monitor by reporting their weight or other body measurements via the website or short message service (SMS) once per week and could view graphs and charts to track their progress overtime. They were also encouraged to self-monitor their dietary intake and exercise using an online diary at least four days per week. The diary provided automated feedback on daily and weekly energy intake and expenditure, and a weekly summary macro- and micronutrient intake compared to recommended targets. Social support was available via a discussion board. Online information was provided weekly (calorie-controlled, low-fat menu plans and grocery lists; physical activity plan based on exercise preferences; educational tips and challenges) which participants were prompted to access via a weekly email newsletter."

"The Enhanced program included all the Basic program features described above. The additional components were: 1) personalized, system-generated enrolment reports that suggested appropriate weight loss goals and key behavior changes required for success based on response to a behavioral survey at enrolment; 2) weekly automated system-generated, personalized e-feedback for key elements of diet and physical activity based on diary entries, usage patterns of website features and level of success with weight loss; 3) an escalating reminder schedule to use the diary, visit the program site and enter a weekly weight (an initial reminder email, then a short message service text message if there was no response, then a reminder phone call if a weekly weight was still not entered)."

5-ix) Describe use parameters

"Participants were encouraged to self-monitor by reporting their weight or other body measurements via the website or short message service (SMS) once per week and could view graphs and charts to track their progress overtime. They were also encouraged to self-monitor their dietary intake and exercise using an online diary at least four days per week."

"...an escalating reminder schedule to use the diary, visit the program site and enter a weekly weight (an initial reminder email, then a short message service text message if there was no response, then a reminder phone call if a weekly weight was still not entered)."

5-x) Clarify the level of human involvement

This was an evaluation of a web-based program, available freely to the public. The only person involvement was at in-person assessments.

5-xi) Report any prompts/reminders used

"Participants were encouraged to self-monitor by reporting their weight or other body measurements via the website or short message service (SMS) once per week and could view graphs and charts to track their progress overtime. They were also encouraged to self-monitor their dietary intake and exercise using an online diary at least four days per week."

"Online information was provided weekly (calorie-controlled, low-fat menu plans and grocery lists; physical activity plan based on exercise preferences; educational tips and challenges) which participants were prompted to access via a weekly email newsletter."
"...an escalating reminder schedule to use the diary, visit the program site and enter a weekly weight (an initial reminder email, then a short message service text message if there was no response, then a reminder phone call if a weekly weight was still not entered)."

5-xii) Describe any co-interventions (incl. training/support)

"At baseline participants were given instructions to log in and set up their program details. They were also given a company phone number in case they experienced any difficulties in logging in. Participants did not receive any training on program use in order to mirror the commercial program engagement experience and increase external validity."

6a) CONSORT: Completely defined pre-specified primary and secondary outcome measures, including how and when they were assessed

The outcomes are specified clearly in the methods paper
"The methods of this assessor-blinded randomized controlled trial have been published in detail elsewhere.[11]"

"Participant assessments were conducted at the University of Newcastle at baseline, 12 and 24 weeks.[11] Blinded research assistants conducted assessments for all groups and participants were reminded at each assessment not to discuss group allocation."
Details of how the outcomes were assessed are also included in the methods section of the manuscript.

6a-i) Online questionnaires: describe if they were validated for online use and apply CHERRIES items to describe how the questionnaires were designed/deployed

Online questionnaires were not used. In-person assessments were carried out.

6a-ii) Describe whether and how "use" (including intensity of use/dosage) was defined/measured/monitored

The number of days participants logged on to the website was recorded.

The previously published methods paper also details how 'usage' was recorded ("Objective measurement of participants' use of the website features (e.g. log-ins to specific program features, diary entries, post to bulletin boards) was collected and stored by SP Health Pty Ltd throughout Phase 1 and Phase 2.")

6a-iii) Describe whether, how, and when qualitative feedback from participants was obtained

Although qualitative feedback was not an outcome measure reported in the current paper, the previously publihsed methods paper details the qualitiative assessment used: ("A process evaluation questionnaire was developed from a previous study [22] to assess the use of website features and satisfaction with the web based program, and completed by participants after 3, 6 and 18-months.")

6b) CONSORT: Any changes to trial outcomes after the trial commenced, with reasons

There was no change to the trial outcomes after trial commencement.

7a) CONSORT: How sample size was determined

7a-i) Describe whether and how expected attrition was taken into account when calculating the sample size

The sample size is detailed in the previously published methods paper ("Based on having 90% power to detect a significant difference in BMI between weight loss groups of 1.5 kg/m2, assuming the SD of BMI is 1.5 and using a two-sided significance level of 0.05, a sample size of 48 participants (24 males and 24 females) is needed for each maintenance group at 18 months. This sample size is within the range of other web-based weight loss intervention studies [13].") In the current study, approximately triple the number of participants were recruited and retained.

7b) CONSORT: When applicable, explanation of any interim analyses and stopping guidelines

Interim analyses were not addressed.

8a) CONSORT: Method used to generate the random allocation sequence

"...randomized using a stratified block design to either the standard (Basic, B) web-based weight loss program or the same program with additional features (Enhanced, E) (Figure 1)."

This information is also detialed in the previously published methods paper ("Randomization
Once written consent was obtained and baseline assessments completed participants were randomly allocated to one of the three Phase 1 groups (Figure 1) using a stratified randomized block design. Participants were stratified by gender and category of body mass index (25 to <30; ≥30 to <35 or ≥35 to 40) using blocks of variable length (either 3 or 6). Subjects received the next envelope in the sequence based on their stratified BMI and gender group. Envelopes were prepared ahead of time, sealed and distributed by a researcher not involved in data collection. Subjects were asked to not open their allocation envelope until they returned home. Subjects were re-randomized in the same way at the end of the weight loss phase to one of the two maintenance arms.")

8b) CONSORT: Type of randomisation; details of any restriction (such as blocking and block size)

"...randomized using a stratified block design to either the standard (Basic, B) web-based weight loss program or the same program with additional features (Enhanced, E) (Figure 1)."

This information is also detialed in the previously published methods paper ("Randomization
Once written consent was obtained and baseline assessments completed participants were randomly allocated to one of the three Phase 1 groups (Figure 1) using a stratified randomized block design. Participants were stratified by gender and category of body mass index (25 to <30; ≥30 to <35 or ≥35 to 40) using blocks of variable length (either 3 or 6). Subjects received the next envelope in the sequence based on their stratified BMI and gender group. Envelopes were prepared ahead of time, sealed and distributed by a researcher not involved in data collection. Subjects were asked to not open their allocation envelope until they returned home. Subjects were re-randomized in the same way at the end of the weight loss phase to one of the two maintenance arms.")

9) CONSORT: Mechanism used to implement the random allocation sequence (such as sequentially numbered containers), describing any steps taken to conceal the sequence until interventions were assigned

"...randomized using a stratified block design to either the standard (Basic, B) web-based weight loss program or the same program with additional features (Enhanced, E) (Figure 1)."

This information is also detialed in the previously published methods paper ("Randomization
Once written consent was obtained and baseline assessments completed participants were randomly allocated to one of the three Phase 1 groups (Figure 1) using a stratified randomized block design. Participants were stratified by gender and category of body mass index (25 to <30; ≥30 to <35 or ≥35 to 40) using blocks of variable length (either 3 or 6). Subjects received the next envelope in the sequence based on their stratified BMI and gender group. Envelopes were prepared ahead of time, sealed and distributed by a researcher not involved in data collection. Subjects were asked to not open their allocation envelope until they returned home. Subjects were re-randomized in the same way at the end of the weight loss phase to one of the two maintenance arms.")

10) CONSORT: Who generated the random allocation sequence, who enrolled participants, and who assigned participants to interventions

Recruitment and enrollment was conducted by researhc personnel. However, the randomisation was prepared and distributed by a researcher not involved in data collection. These randomisation codes were also not known to the distributor as they were concealed in envelopes.

This information is also detailed in the previously published methods paper ("Randomization...Envelopes were prepared ahead of time, sealed and distributed by a researcher not involved in data collection.")

11a) CONSORT: Blinding - If done, who was blinded after assignment to interventions (for example, participants, care providers, those assessing outcomes) and how

11a-i) Specify who was blinded, and who wasn't

Both participants and research personnel were blinded to group allocation.

"Participants were blinded to group allocation,..."

"Blinded research assistants conducted assessments for all groups and participants were reminded at each assessment not to discuss group allocation."

11a-ii) Discuss e.g., whether participants knew which intervention was the "intervention of interest" and which one was the "comparator"

Participants were blinded to group allocation and were unaware whether their program was wioth or without additional features.
"Participants were blinded to group allocation,..."

11b) CONSORT: If relevant, description of the similarity of interventions

The paper indicates the similarities and differences between the two programs.

"The Enhanced program included all the Basic program features described above. The additional components were: 1) personalized, system-generated enrolment reports that suggested appropriate weight loss goals and key behavior changes required for success based on response to a behavioral survey at enrolment; 2) weekly automated system-generated, personalized e-feedback for key elements of diet and physical activity based on diary entries, usage patterns of website features and level of success with weight loss; 3) an escalating reminder schedule to use the diary, visit the program site and enter a weekly weight (an initial reminder email, then a short message service text message if there was no response, then a reminder phone call if a weekly weight was still not entered)."

12a) CONSORT: Statistical methods used to compare groups for primary and secondary outcomes

"Analysis of covariance (ANCOVA) was used to test for differences in outcomes at 12 weeks and 24 weeks between treatment groups after adjusting for the baseline value of that outcome. The model outcome was the variable of interest at 12 or 24 weeks with the baseline level used as a covariate. The only other variable included in the model was sex. Differences and 95% confidence intervals between treatment groups in the outcome at each time point were estimated using the least square means from the ANCOVA models."

"An additional analysis was conducted using a generalized linear mixed model (GLMM) to test for a difference between groups across the combined 12 week and 24 week time points."

12a-i) Imputation techniques to deal with attrition / missing values

"The Intention to Treat (ITT) population includes all subjects who were randomized into one of the two treatment groups. For subjects who had missing data at 12 or 24 weeks their missing data was imputed using the Last Observation Carried Forward (LOCF) approach. The completer population (CP) includes all individuals who attended the 24 week assessment and subgroup analyses are based on this population."

12b) CONSORT: Methods for additional analyses, such as subgroup analyses and adjusted analyses

Analyses were adjusted for sex and baseline value for the specified outcome.

RESULTS

13a) CONSORT: For each group, the numbers of participants who were randomly assigned, received intended treatment, and were analysed for the primary outcome

"Of the 591 participants assessed for eligibility, 309 (129 males) were initially randomized into the three groups (Basic n=99, Enhanced n= 106 or wait-list control n= 104). After 12 weeks the controls, of whom eight were lost to follow-up, were re-randomized (96 participants, 52 Enhanced, 44 Basic) into the trial. Therefore, in the current analysis 301 participants (125 males) were randomized to the (Basic (n=143) or Enhanced n=158) groups (Figure 1)."

13b) CONSORT: For each group, losses and exclusions after randomisation, together with reasons

Figure 1 details the reasons for losses and exclusions.

"Of the 591 participants assessed for eligibility, 309 (129 males) were initially randomized into the three groups (Basic n=99, Enhanced n= 106 or wait-list control n= 104). After 12 weeks the controls, of whom eight were lost to follow-up, were re-randomized (96 participants, 52 Enhanced, 44 Basic) into the trial. Therefore, in the current analysis 301 participants (125 males) were randomized to the (Basic (n=143) or Enhanced n=158) groups (Figure 1)."

13b-i) Attrition diagram

A figure was not provided for this reason. However, attrition rates and website usage rates are reported in text.

"There was no significant difference in retention rates between the Basic (74.7%) and Enhanced (84.9%) groups after 12-weeks (p=0.66), however more Enhanced group participants attended the 24-week assessments (B 69%, E 81%, p=0.012)."

"There was a significantly greater website usage in the Enhanced group compared to the Basic group at both 12 and 24 weeks with the Enhanced group logging on an additional 10 days over the first 12 weeks and 12 days over 24 weeks (p<0.002) (Table 4)."

14a) CONSORT: Dates defining the periods of recruitment and follow-up

Recruitment and follow-up periods are detailed in the previously published methods paper.

14a-i) Indicate if critical "secular events" fell into the study period

No problems occured.

14b) CONSORT: Why the trial ended or was stopped (early)

The trial was not ended prematurely

15) CONSORT: A table showing baseline demographic and clinical characteristics for each group

Baseline characteristics are included in Table 1 and referred to in text.

"Baseline characteristics
Of the 591 participants assessed for eligibility, 309 (129 males) were initially randomized into the three groups (Basic n=99, Enhanced n= 106 or wait-list control n= 104). After 12 weeks the controls, of whom eight were lost to follow-up, were re-randomized (96 participants, 52 Enhanced, 44 Basic) into the trial. Therefore, in the current analysis 301 participants (125 males) were randomized to the (Basic (n=143) or Enhanced n=158) groups (Figure 1). Subjects who were randomized to the Basic WL group were similar at baseline to those randomized to the Enhanced WL group for all demographic and other baseline characteristics (Table 2). Participants were approximately 42 years old, overweight (36%) or obese category 1 (40%), Australian born (91%), and 70% of participants reported weekly household incomes of ≥AUS$1500."

15-i) Report demographics associated with digital divide issues

"Baseline characteristics
Of the 591 participants assessed for eligibility, 309 (129 males) were initially randomized into the three groups (Basic n=99, Enhanced n= 106 or wait-list control n= 104). After 12 weeks the controls, of whom eight were lost to follow-up, were re-randomized (96 participants, 52 Enhanced, 44 Basic) into the trial. Therefore, in the current analysis 301 participants (125 males) were randomized to the (Basic (n=143) or Enhanced n=158) groups (Figure 1). Subjects who were randomized to the Basic WL group were similar at baseline to those randomized to the Enhanced WL group for all demographic and other baseline characteristics (Table 2). Participants were approximately 42 years old, overweight (36%) or obese category 1 (40%), Australian born (91%), and 70% of participants reported weekly household incomes of ≥AUS$1500."

16a) CONSORT: For each group, number of participants (denominator) included in each analysis and whether the analysis was by original assigned groups

16-i) Report multiple “denominators” and provide definitions

The number of people included in each analyses is included in the tables and in text.

16-ii) Primary analysis should be intent-to-treat

"The Intention to Treat (ITT) population includes all subjects who were randomized into one of the two treatment groups."

17a) CONSORT: For each primary and secondary outcome, results for each group, and the estimated effect size and its precision (such as 95% confidence interval)

"Retention at 12 and 24 weeks
Participant flow through the trial (Figure 1) shows the number of participants who were randomized to each treatment condition, the number who withdrew with reasons, and the number who had data at 12- and 24-weeks. There was no significant difference in retention rates between the Basic (74.7%) and Enhanced (84.9%) groups after 12-weeks (p=0.66), however more Enhanced group participants attended the 24-week assessments (B 69%, E 81%, p=0.012)."

17a-i) Presentation of process outcomes such as metrics of use and intensity of use

"Website usage
There was a significantly greater website usage in the Enhanced group compared to the Basic group at both 12 and 24 weeks with the Enhanced group logging on an additional 10 days over the first 12 weeks and 12 days over 24 weeks (p<0.002) (Table 4). A similar result was found for the completers population (Table 4)."

17b) CONSORT: For binary outcomes, presentation of both absolute and relative effect sizes is recommended

Binary outcomes are not assessed.

18) CONSORT: Results of any other analyses performed, including subgroup analyses and adjusted analyses, distinguishing pre-specified from exploratory

"Subgroup analyses
The change in primary and secondary outcomes within treatment groups was similar across all subgroups (sex, age, BMI category) of the completer population 12 or 24 weeks (data not presented). There were no statistically significant interactions between treatment group and sex (p=0.52), treatment group and BMI category (p=0.45) or treatment group and age group (p=0.72) for the outcome of weight."

18-i) Subgroup analysis of comparing only users

Both ITT and completers only was assessed.

19) CONSORT: All important harms or unintended effects in each group

Unintended effects/ hamrs did not occur.

19-i) Include privacy breaches, technical problems

Technical or privacy breaches did not occur.

19-ii) Include qualitative feedback from participants or observations from staff/researchers

Qualitative feedback was collected but is not included in the manuscript.

DISCUSSION

20) CONSORT: Trial limitations, addressing sources of potential bias, imprecision, multiplicity of analyses

20-i) Typical limitations in ehealth trials

"A limitation of the current study is that it did not have a wait list control group at six months."
"Attrition reduced the power to detect significant differences between groups, particularly for the secondary outcomes.."

21) CONSORT: Generalisability (external validity, applicability) of the trial findings

21-i) Generalizability to other populations

This is not addressed explicitly but the participants were recruited from the general community with no exclusion for computer literacy. The study is designed to test external validity.

"Participants did not receive any training on program use in order to mirror the commercial program engagement experience and increase external validity."

21-ii) Discuss if there were elements in the RCT that would be different in a routine application setting

THis was not applicable as the programs were commercially available and would be available in the general setting.

22) CONSORT: Interpretation consistent with results, balancing benefits and harms, and considering other relevant evidence

22-i) Restate study questions and summarize the answers suggested by the data, starting with primary outcomes and process outcomes (use)

the opening paragraph is as follows:
"The aim study was to evaluate whether overweight and obese adults randomized to a commercial web-based weight loss program providing greater social support and more personalized feedback achieved a greater reduction in BMI and increased usage of program features compared to those randomized to a standard version of the program. We found no differences in weight loss or a host of secondary health outcomes between the Basic and Enhanced features versions of the web-based weight loss program after 24 weeks, despite previous reports that provision of enhanced features within web-based formats does enhance weight loss outcomes [6]. With mean weight loss in the current study ranging from 3.6% to 4.3% in both intervention groups after 24 weeks, both versions look promising at the population level...When we examined those who achieved ≥5% weight loss, success was strongly associated with website usage, indicating that strategies to improve website usage may be beneficial to weight loss outcomes. In this regard, some aspects of the Enhanced program features may be valuable, as the Enhanced group had a significantly lower dropout rate and greater participant engagement."

22-ii) Highlight unanswered new questions, suggest future research

"Future programs may need to segment the target population in order to improve feedback tailoring to specific user groups as a strategy to avoid website discontinuity, particularly in relation to some age, sex or BMI sub-groups.[26] Some groups may not need this more extensive feedback and it would be useful to identify who they are."

"Further research examining which combination of website features optimize program use and reduce attrition are needed. Based on the current study, future modifications to the Enhanced program would need to achieve a 50 % increase in the number of participant logins than that in the current study. This would mean getting participants to use the program at least 2-3 times per week as a strategy to facilitate clinically important ≥5% weight loss. Establishing and testing these targets could ease the burden and fatigue associated with program usage targets that are not achievable or sustainable."

"We cannot tell whether the reminders schedule to login and use of program features in the current study was the key driver of this and this also needs to be examined in future studies."

Other information

23) CONSORT: Registration number and name of trial registry

"Australian New Zealand Clinical Trials Registry (ANZCTR) http://www.anzctr.org.au/ Trial number: ACTRN12610000197033"

24) CONSORT: Where the full trial protocol can be accessed, if available

"The methods of this assessor-blinded randomized controlled trial have been published in detail elsewhere.[11]"
"Collins CE, Morgan PJ, Jones P, Fletcher K, Martin J, Aguiar EJ, et al. Evaluation of a commercial web-based weight loss and weight loss maintenance program in overweight and obese adults: a randomised controlled trial. BMC Public Health. 2010;10:669."

25) CONSORT: Sources of funding and other support (such as supply of drugs), role of funders

"Sources of support:
This trial was funded by an Australian Research Council Linkage Project grant (2009-2012) (LP0990414, G0189752), with SP Health as the Industry Partner Organization (G0189753). CEC is supported by a National Health and Medical Research Council Australian Career Development Fellowship (#6315005). MN is supported by a Post-doctoral fellowship from the Priority Research Centre in Physical Activity and Nutrition, The University of Newcastle"

X26-i) Comment on ethics committee approval

"Written informed consent was obtained from all participants, and ethical approval obtained from the University of Newcastle Human Ethics Research Committee."

x26-ii) Outline informed consent procedures

"Written informed consent was obtained from all participants, and ethical approval obtained from the University of Newcastle Human Ethics Research Committee."

X26-iii) Safety and security procedures

"At baseline participants were given instructions to log in and set up their program details. They were also given a company phone number in case they experienced any difficulties in logging in."

X27-i) State the relation of the study team towards the system being evaluated

"CONFLICTS OF INTEREST
This trial was funded by an Australian Research Council Linkage Project grant (2009–2012) (LP0990414, G0189752), with SP Health as the Industry Linkage Partner Organization (G0189753).
CEC has been a nutrition consultant to SP Health Co. M Hutchesson (nee Neve) was funded by a Penn Health post-doctoral fellowship. All other authors declare that they have no competing interests."
