# Supplementary figures and images for: Efficacy of Standard Versus Enhanced Features in a Web-Based Commercial Weight-Loss Program for Obese Adults, Part 2: Randomized Controlled Trial
Source: J Med Internet Res. 2013 Jul 22;15(7):e140. doi: 10.2196/jmir.2626 (PMC3786000; doi:10.2196/jmir.2626)

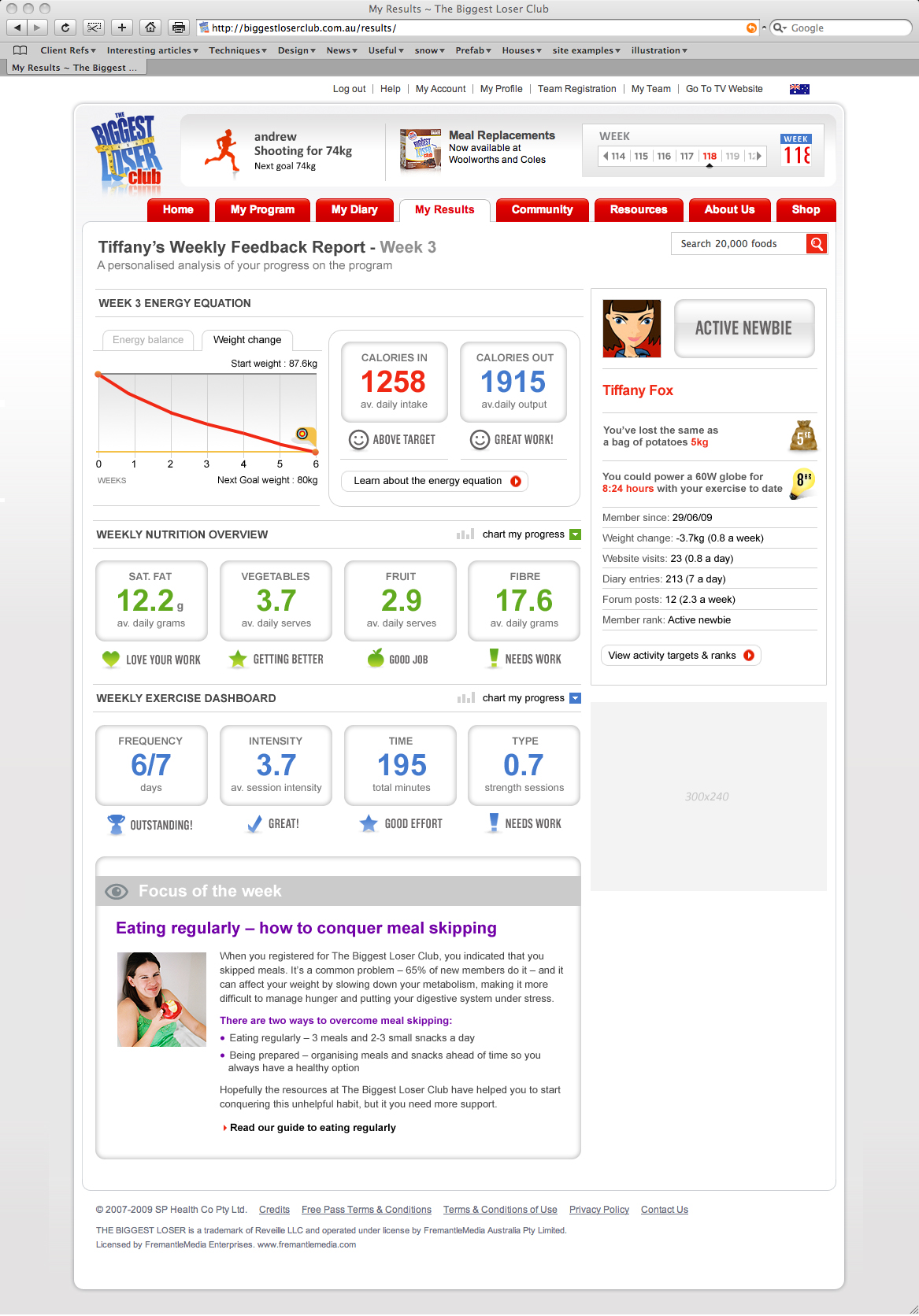

Supplement: Supplementary file 2 [file jmir_v15i7e140_app2.jpg]

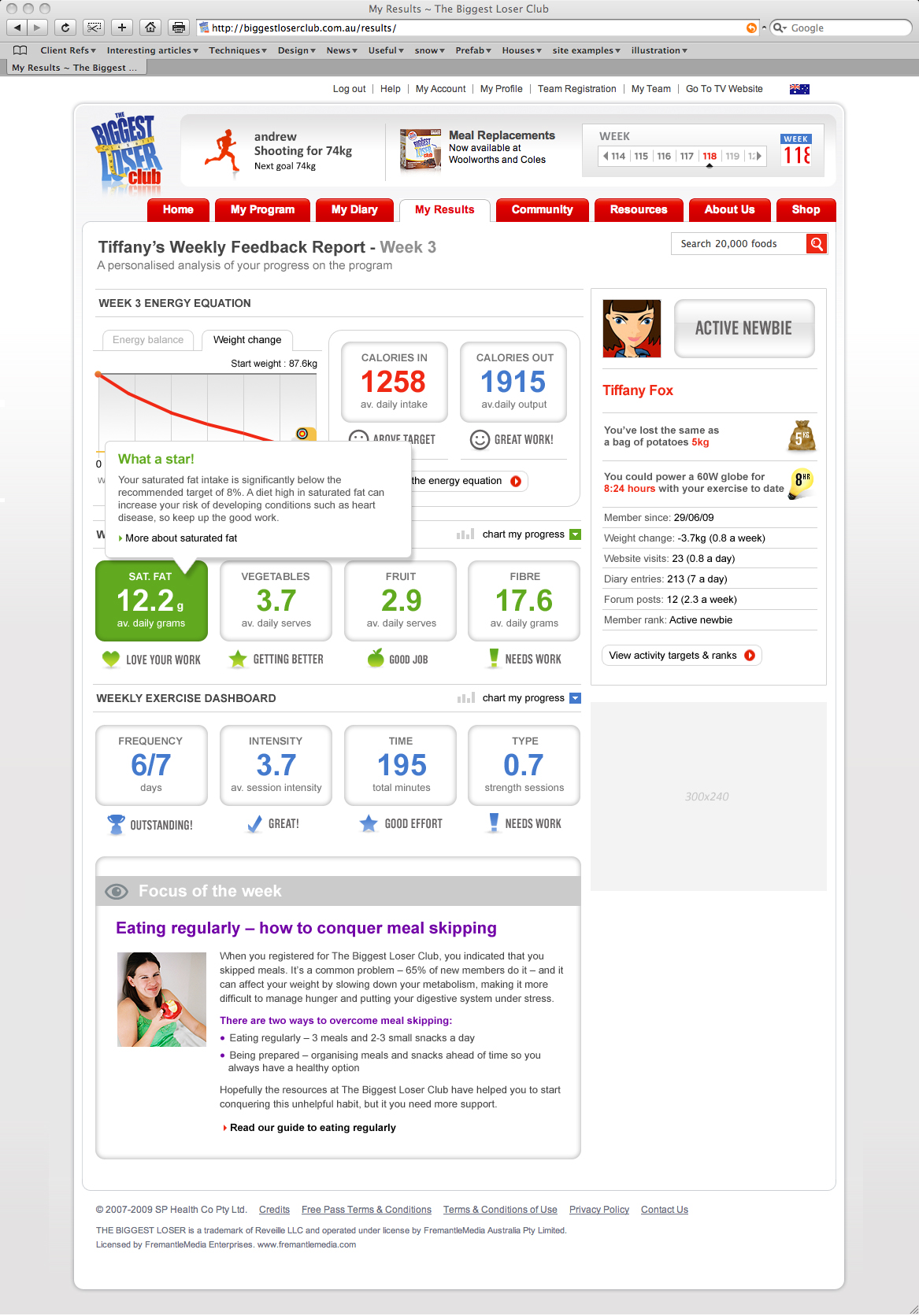

Supplement: Supplementary file 3 [file jmir_v15i7e140_app3.jpg]
